# The complete mitochondrial genome data of *Ylistrum balloti* (Bernardi 1861) (Bivalvia: Pectinidae) from China

**DOI:** 10.1016/j.dib.2025.112437

**Published:** 2026-01-07

**Authors:** Dewei Cheng, Fangchao Zhu, Lintao Zhao, Ying Qiao, Ersha Dang, Xuyang Chen

**Affiliations:** a Key Laboratory of Tropical Marine Ecosystem and Bioresource, Fourth Institute of Oceanography, Ministry of Natural Resources, 536015, Beihai, China; b Guangxi Key Laboratory of Beibu Gulf Marine Resources, Environment and Sustainable Development, Fourth Institute of Oceanography, Ministry of Natural Resources, 536015, Beihai, China; c Observation and Research Station of Coastal Wetland Ecosystem in Beibu Gulf, Ministry of Natural Resources, 536015, Beihai, China

**Keywords:** *Ylistrum balloti*, Mitochondrial genome, Phylogenetic analysis, Marine organism

## Abstract

The marine bivalve *Ylistrum balloti*, an economically important species found in the South China Sea, remains largely unexplored in terms of its genetic background. In this study, we have determined the complete mitochondrial genome of *Y. balloti*. The entire mitogenome of *Y. balloti* spans 19484 bp, with a base composition of 21.94% A, 13.12% C, 29% G and 35.94% T. The genome contains 13 protein-coding genes (PCGs), 3 ribosomal RNA (rRNA) genes, 23 transfer RNA (tRNA) genes, and a major non-coding region (MNR). A phylogenetic analysis, based on 12 protein-coding genes (PCGs) from 15 species within related taxa, supported the grouping of *Y. balloti* with *Y. japonicum* and their clustering within the Pectinidae family. This dataset presents the complete mitochondrial genome of *Y. balloti*, providing insights for studies in evolution, taxonomy, DNA barcoding, and population genetics of *Ylistrum*.

Specifications Table

**Table:** 

Subject	Biology
Specific subject area	Mitogenomics and Phylogeny
Type of data	Table, Figure, GenBank, Fasta
Data collection	Genomic DNA source: An individual specimen Genomic DNA isolation: Genomic DNA Isolation Kit (QiaGen, Beijing, China) Library construction: TruSeqTM Nano DNA Sample Prep Kit, Illumina Sequencing: Illumina NovaSeq 6000 platform (150 bp paired- end; Illumina, San Diego,CA, USA) Quality check: Illumina Experiment Manager, MiSeq Reporter Assembly and annotation: SPAdes version 3.11.0, MITOS2 webserver Mitogenome map construction: ProkSee. (https://proksee.ca/) Phylogenetic tree analysis: MEGA v.11.0 tRNA analysis: tRNAScan-SE 2.0 Phylogenetic tree visualization: iTOL (https://itol.embl.de/)
Data source location	Institution:Fourth Institute of Oceanography, Ministry of Natural Resources‌ City/Town/Region: Beihai, Guangxi Country: China Latitude and longitude (and GPS coordinates, if possible) for collected samples/data: Latitude: 21°20’24’’ N, Longitude: 109°22’53’’ E)
Data accessibility	Repository name: NCBI Sequence Read Archive (SRA) Data identification number: SRR18579608 Direct URL to data: https://www.ncbi.nlm.nih.gov/sra/SRR18579608 Repository name: NCBI BioProject Data identification number: PRJNA821604 Direct URL to data: https://www.ncbi.nlm.nih.gov/bioproject/PRJNA821604 Repository name: NCBI BioSample Data identification number: SAMN27116001 Direct URL to data: https://www.ncbi.nlm.nih.gov/biosample/SAMN27116001 Repository name: NCBI GenBank Data identification number: ON041136 Direct URL to data: https://www.ncbi.nlm.nih.gov/nuccore/ON041136
Related research article	Not applicable

## 1 Value of the Data

• The genomic data provide a crucial molecular reference for resolving the current taxonomic uncertainties surrounding Y. balloti, helping to establish its precise systematic position.
• These data offer valuable information for understanding phylogenetic relationships and evolutionary patterns within the Pectinidae family, contributing to broader studies of bivalve evolution.
• The genetic resource generated in this study will facilitate evidence-based conservation strategies and sustainable fisheries management of this commercially important marine species
• Furthermore, this mitogenome serves as a valuable resource for comparative mitogenomics, DNA barcoding, and phylogeographic studies within the family Pectinidae.

## 2 Background

**Fig. 1:**
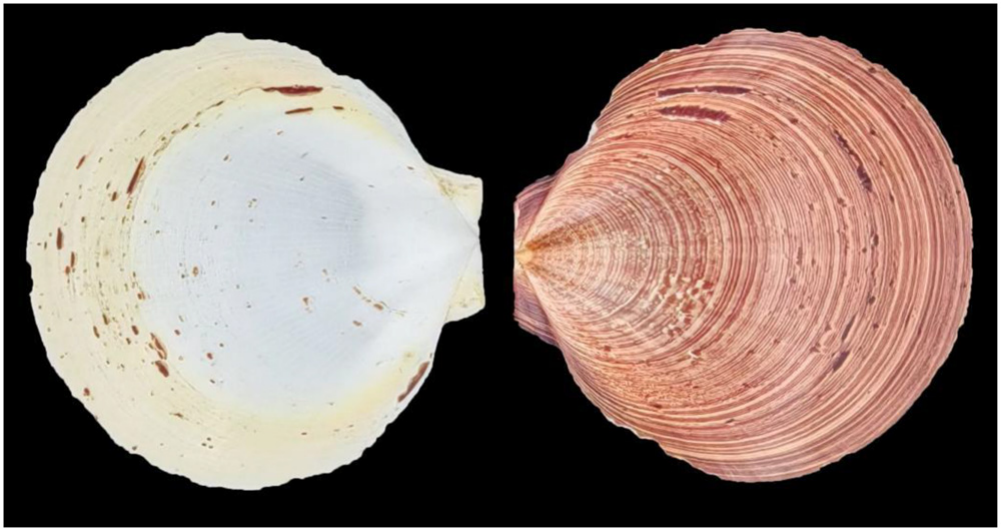
Specimen image of the *Y. balloti*, was collected in November 2021 from Beibu Gulf, China. The image was photographed by Dewei Cheng.

The scallop species *Ylistrum balloti* (Bernardi, 1861) (Bivalvia: Pectinidae) represents a taxonomically challenging and economically important marine bivalve group. Initially described in Chinese waters as *Amusium japonicum balloti* [1], its classification was subsequently revised and assigned to the genus *Ylistrum* based on systematic updates in pectinid taxonomy [2]. This genus includes two recognized species, *Ylistrum japonicum* and *Ylistrum balloti*, which are differentiated by several morphological characteristics. Collected specimens from the Beibu Gulf in the South China Sea exhibit identifying features that align with historical descriptions of *Y. balloti* [1,2], such as distinctive concentric spot patterns and specific valve coloration (Fig. 1). These traits contrast with those of *Y. japonicum*, which typically displays darker auricles and an absence of concentric spots on both valves.

However, the taxonomic status of these Chinese specimens remains uncertain. Recent mitochondrial genome sequencing of *Y. japonicum* shows 98.99% similarity with our specimens [3], indicating a significant discrepancy between morphological and molecular data. This high genetic similarity suggests two plausible scenarios: either (a) the Chinese and Japanese/Australian populations represent very recently diverged sister species or subspecies, or (b) they may be conspecific, with the observed morphological differences attributable to ecophenotypic plasticity or population-level variation. Additionally, the known distribution of *Y. balloti* is mainly documented in Australia’s western, eastern, and southern regions [4], with no genetically confirmed occurrences in China. This inconsistency underscores a persistent taxonomic ambiguity. The mitogenome presented here serves as a foundation for integrated taxonomic studies aimed at resolving this species delimitation issue.

To resolve this uncertainty, we report the complete mitochondrial genome of the putative *Y. balloti* obtained from the South China Sea. This genomic resource offers valuable data for clarifying phylogenetic relationships and species delimitation within the genus *Ylistrum* in the region. It provides a critical molecular reference for future phylogenetic studies and supports accurate species identification, which is crucial for both conservation planning and sustainable management of this commercially significant species.

## 3 Data Description

### 3.1 Characteristics of *Y. balloti* genome

**Fig. 2:**
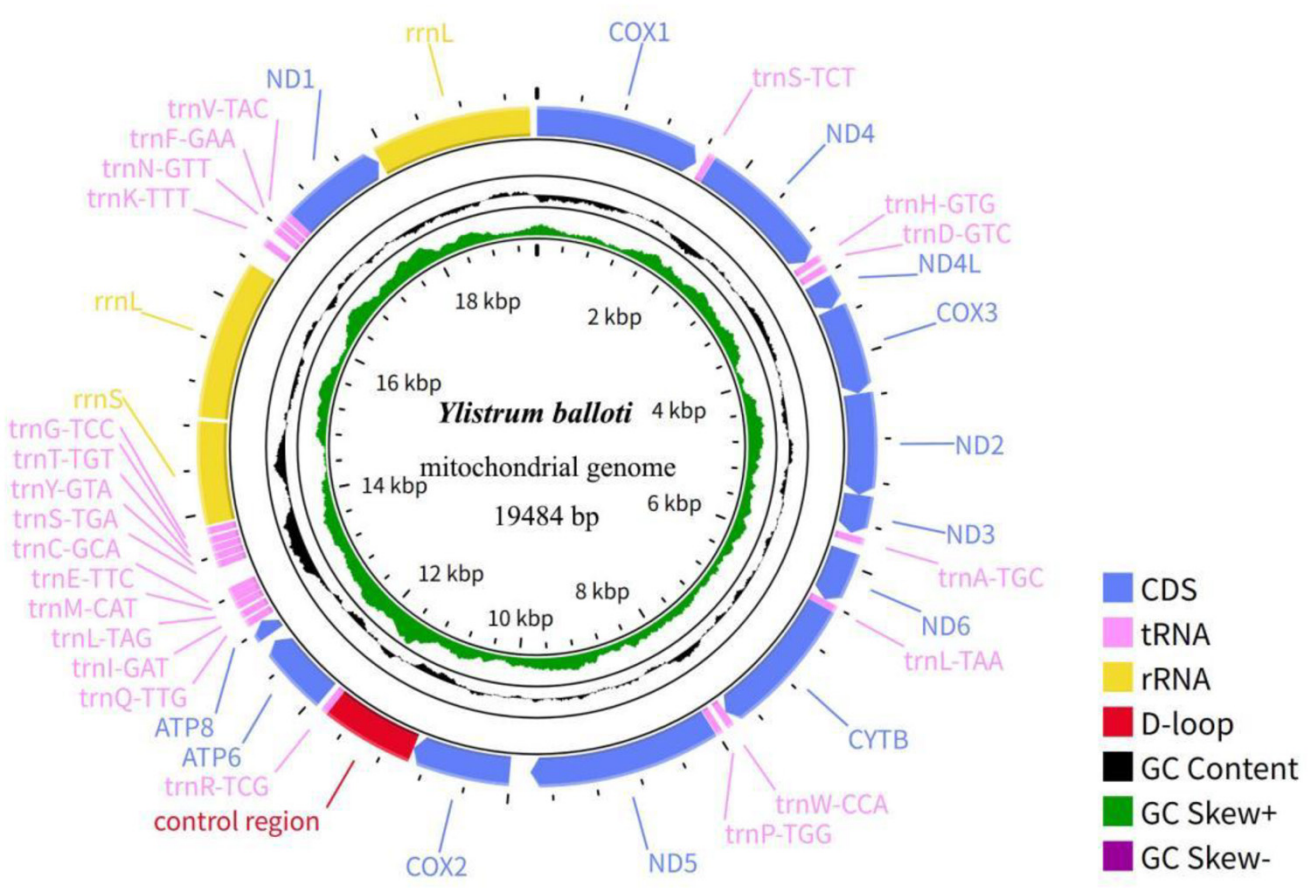
Mitochondrial genome map of *Y. balloti* (GenBank accession number: ON041136). Genes are color-coded according to their functional classification. Blue represents 13 PCGs, pink represents 23 tRNAs, yellow represents 3 rRNAs, red represents control D-loop regions. The black color in the inner ring indicates GC content, while the green and purple represent GC skew. From the figure, it is evident that the GC skew is positive. The coding genes are located on the heavy strand, and arrows indicate the directions of transcription.

The complete mitochondrial genome of *Y. balloti* is 19484 bp in length and comprises 13 protein-coding genes (*COX1, ND4, ND4L, COX3, ND2, ND3, ND6, CYTB, ND5, COX2, ATP6, ATP8, ND1*), 3 rRNA genes (one 12S rRNA and two 16S rRNA genes), 23 tRNA genes and a control region (D-loop) (Fig. 2). Notably, we identified an additional 16S rRNA gene, representing a specific genomic rearrangement also reported in its congener *Y. japonicum* [3], suggesting it may be a shared characteristic within the genus. This duplication was validated by PCR and Sanger sequencing, confirming it as a genuine biological feature (Supplementary Figs. S1-S3). Sequencing on an Illumina NovaSeq 6000 platform yielded 10.24 GB of raw sequencing reads, resulting in a final assembly with 415.79 × coverage depth (Supplementary Fig. S4). The overall base composition of the mitochondrial genome is estimated to be A: 21.94%, T: 35.94%, C: 13.12%, and G: 29%. The 57.88% of (A+T) content shows a strong preference over the 42.12% of (G + C) content, which is similar to the reported mitochondrial genomes of the most marine bivalve mollusks [[5], [6], [7]]. Start codons for the PCGs in *Y. balloti* are ATG, GTG or TTG. Twelve PCGs terminate with the canonical stop codons TAA or TAG, whereas the *CYTB* gene ends with an incomplete stop codon (T). This incomplete codon is presumed to be post-transcriptionally polyadenylated to form TAA, a common mechanism in metazoan mitochondria. The lengths of the 23 tRNA genes range from 65 bp to 72 bp (Table 1). The complete mitogenome sequence has been deposited in GenBank under the accession number of ON041136.

**Table 1:** Characteristics of the mitochondrial genome of *Y. balloti*. Direction indicates the forward strand (+).

Gene	Start (bp)	End (bp)	Length (bp)	Direction	Start/stop codon
COX1	1	1584	1584	+	ATG/TAA
trnS-TCT	1645	1712	68	+	
ND4	1718	3007	1290	+	ATG/TAG
trnH-GTG	3014	3078	65	+	
trnD-GTC	3116	3183	68	+	
ND4L	3231	3515	285	+	GTG/TAA
COX3	3520	4365	846	+	GTG/TAG
ND2	4407	5330	924	+	GTG/TAA
ND3	5330	5701	372	+	ATG/TAA
trnA-TGC	5716	5781	66	+	
ND6	5874	6383	510	+	TTG/TAA
trnL-TAA	6385	6449	65	+	
CYTB	6453	7827	1375	+	TTG/T–
trnW-CCA	7828	7896	69	+	
trnP-TGG	7947	8011	65	+	
ND5	8019	9806	1788	+	TTG/TAG
COX2	9864	10949	1086	+	ATG/TAG
D-loop	10950	11810	861	+	
trnR-TCG	11811	11878	68	+	
ATP6	11929	12642	714	+	ATG/TAG
trnM-TAT	12647	12711	65	+	
ATP8	12715	12870	156	+	GTG/TAG
trnQ-TTG	12882	12952	71	+	
trnI-GAT	12973	13043	71	+	
trnL-TAG	13069	13137	69	+	
trnM-CAT	13144	13215	72	+	
trnE-TTC	13219	13284	66	+	
trnC-GCA	13485	13550	66	+	
trnS-TGA	13561	13627	67	+	
trnY-GTA	13637	13703	67	+	
trnT-TGT	13715	13782	68	+	
trnG-TCC	13804	13869	66	+	
rrnS	13891	14847	957	+	
rrnL1	14885	16371	1487	+	
trnK-TTT	16579	16650	72	+	
trnN-GTT	16735	16801	67	+	
trnF-GAA	16815	16879	65	+	
trnV-TAC	16894	16960	67	+	
ND1	16963	17910	948	+	GTG/TAG
rrnL2	17928	19419	1492	+	

### 3.2 Phylogenetic analysis

**Fig. 3:**
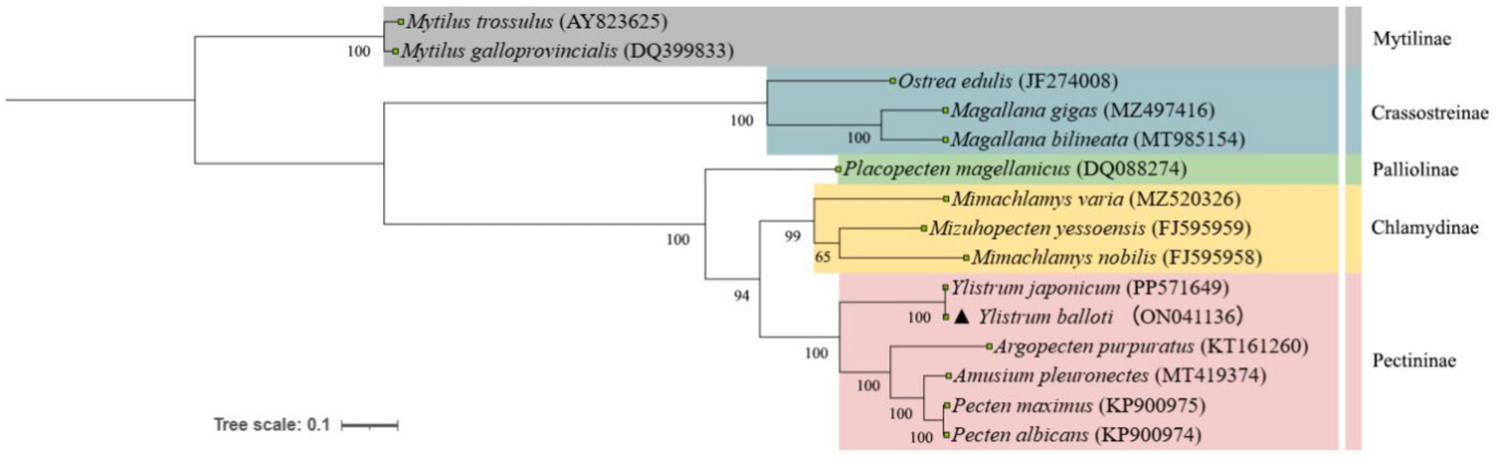
Maximum-likelihood (ML) phylogenetic tree was reconstructed based on the concatenated 12 protein-coding genes of *Y. balloti* and other 14 species. Accession numbers were indicated after the species name. Numbers at the nodes indicated bootstrap support value from 1000 replicates.

A maximum-likelihood phylogenetic tree was constructed using 12 PCGs (excluding *ATP8*) from *Y. balloti* and 14 related species sourced from GenBank (Fig. 3). The results showed that *Y. balloti* and *Y. japonicum* formed a maximally supported clade (bootstrap value =100), which was sister to a clade comprising *Argopecten purpuratus, Amusium pleuronectes, Pecten albicans*, and *Pecten maximus*. These relationships are congruent with previous phylogenetic studies of Pectinidae [8] and confirm the close sister-species relationship between *Y. balloti* and *Y. japonicum* at the molecular level.

Thus, while our mitogenomic data clearly delineates the phylogenetic position of *Y. balloti* within Pectinidae and recovers its robust sister-species relationship with *Y. japonicum*, the extreme genetic similarity between them (as noted in the Background) highlights a persistent taxonomic ambiguity. This dataset therefore serves as a critical molecular reference, and definitive species delimitation within *Ylistrum* will require future integrative taxonomic approaches incorporating nuclear genomic markers and broader population sampling.

## 4 Experimental Design, Materials and Methods

### 4.1 Sample collection

The sample of *Y. balloti* was collected in November 2021 from Beibu Gulf, China (109°22’53’’ E, 21°20’24’’ N) by commercial trawling, and deposited in the specimen room of Fourth Institute of Oceanography, Ministry of Natural Resources (contact person: DW-Cheng, email: dwcheng2022@163.com) with voucher number Aj20211103).

### 4.2 DNA extraction and sequencing

Total genomic DNA was extracted from gill tissues using a Genomic DNA Isolation Kit (QiaGen, Beijing, China), following the manufacturer’s protocols [9]. After isolating the DNA, 1 µg sample DNA was fragmented to a size of 300-500 bp using the Covaris M220 system [10]. Short-insert libraries were constructed according to the manufacturer’s instructions (TruSeqTM Nano DNA Sample Prep Kit, Illumina) and sequenced on an Illumina NovaSeq 6000 platform (llumina Inc, San Diego, CA, USA) with 150 bp paired-end reads [11,12].

### 4.3 Assembly, annotation, and analysis

Raw sequencing data were quality-filtered using fastp with the following criteria: removal of reads containing >5% N bases, those with >50% of bases having a Phred quality score ≤5, and adapter sequences. This produced approximately 9.83 Gb of clean data (Q20 = 96.68%, Q30 = 92.09%). The clean reads were assembled *de novo* using SPAdes (v.3.11.0) [13] with multiple k-mer sizes (21, 45, 65, 85, 105). The complete mitochondrial genome was identified and extracted from the assembly graph based on coverage depth and circularity. Base composition and GC/AT skew were estimated by using BioEdit [14]. Transfer RNA (tRNA) genes were predicted using the on-line software tRNAScan-SE (v.2.0) [15]. The genome was annotated using MITOS [16], with manual curation of the automated output. The final annotated genome map was drawn using Proksee [17].

### 4.4 Phylogenetic analysis

For precise determination of *Y. balloti* phylogenetic position, a comparative analysis has been executed by utilizing the concatenated amino acid sequences of 12 mitochondrial PCGs (excluding *ATP8*) from 15 species across five families, to avoid potential issues arising from its short length and variable presence among taxa. These sequences were sourced from the NCBI GenBank database for an efficient assessment. Multiple sequence alignment of the concatenated PCGs was performed with Clustal W in MEGA11 (v.11.0.13) [18]. The mtREV+G+I+F model was selected as the optimal substitution model. Maximum Likelihood (ML) trees were reconstructed under this model, with branch support evaluated by 1,000 bootstrap replicates. The tree was rooted with *Mytilus trossulus* and *Mytilus galloprovincialis* as the outgroup [3]. Final visualization and annotation were conducted on the iTOL platform (https://itol.embl.de).

### 4.5 PCR Validation of the 16S rRNA Duplication

To verify the duplicated 16S rRNA genes identified in the assembly, PCR validation was conducted using six primer pairs (Supplementary Table 1) designed to specifically amplify the junction regions spanning the two 16S rRNA gene copies and their flanking sequences. PCR was performed using 20 ng of genomic DNA in a 30 µL reaction volume containing 2 × Taq PCR Master Mix and 10 pmol of each primer. Thermal cycling conditions were: initial denaturation at 95°C for 5 min; 35 cycles of 95°C for 30 s, 60°C for 30 s, and 72°C for 45 s; final extension at 72°C for 5 min. PCR products were separated by 1% agarose gel electrophoresis to confirm specific amplification. The resulting amplicons of expected sizes were purified and subjected to Sanger sequencing on an ABI 3730xl DNA Analyzer. The resulting sequences were aligned against the assembled mitogenome using ClustalW, and the alignments were visualized with GeneDoc software to verify their identity and the precise junction regions. Detailed validation results are provided in Supplementary Figs. S1–S3.

## Limitations

Not applicable.

## Ethics Statement

The authors have read and followed the ethical requirements for publication in Data in Brief. The authors confirm the current work does not involve human subjects, animal experiments, or any data collected from social media platforms.

## CRediT Author Statement

**Dewei Cheng:** Conceptualization, data curation, Formal analysis, Investigation, Writing-original draft; **Fangchao Zhu:** Data curation, Investigation, Methodology; **Lintao Zhao:** Investigation, Validation; **Ying Qiao:** Resources, Investigation; **Ersha Dang** and **Xuyang Chen:** Conceptualization, Funding acquisition, Project administration, Resources, Supervision, Validation, Writing- review & editing. All authors have approved the final version of manuscript and all authors agree to be accountable for all aspects of the work.

## Data Availability

NCBINCBI Sequence Read Archive (SRA) (Original data).

## Appendix Supplementary materials

Image, application 1
