# Supplementary material for: The complete mitochondrial genome data of Ylistrum balloti (Bernardi 1861) (Bivalvia: Pectinidae) from China
Source: Data Brief. 2026 Jan 7;64:112437. doi: 10.1016/j.dib.2025.112437 (PMC12855584; doi:10.1016/j.dib.2025.112437)
Supplement: Supplementary file 1 [file mmc1.docx]

**Supplementary Fig. S1. Validation of the first 16S rRNA copy (rrnL1).** The Sanger sequence from an internal amplicon aligns perfectly with the first 16S rRNA gene in the assembly (ON041136), confirming its nucleotide sequence.


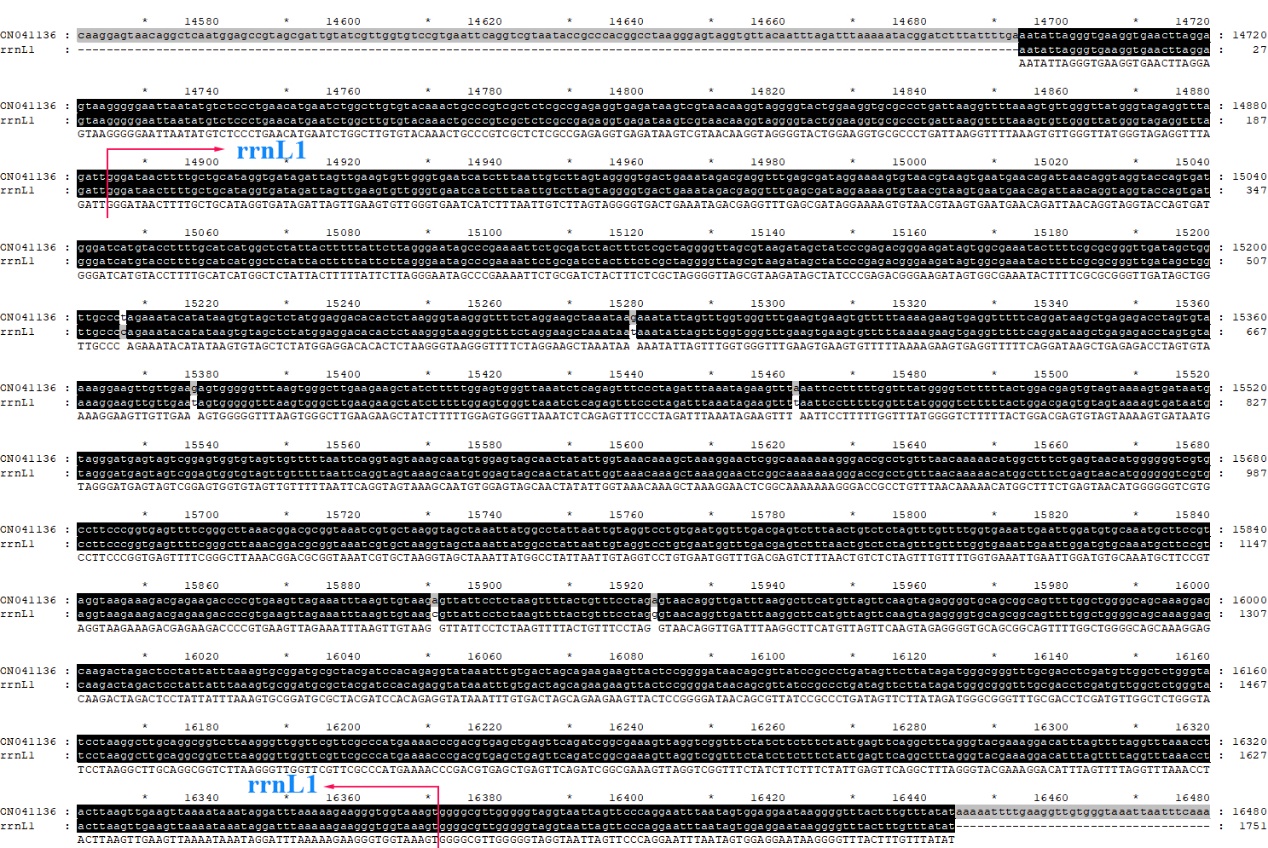


**Supplementary Fig. S2. Validation of the second 16S rRNA copy (rrnL2).** The Sanger sequence from an internal amplicon aligns perfectly with the second 16S rRNA gene in the assembly, confirming its nucleotide sequence.


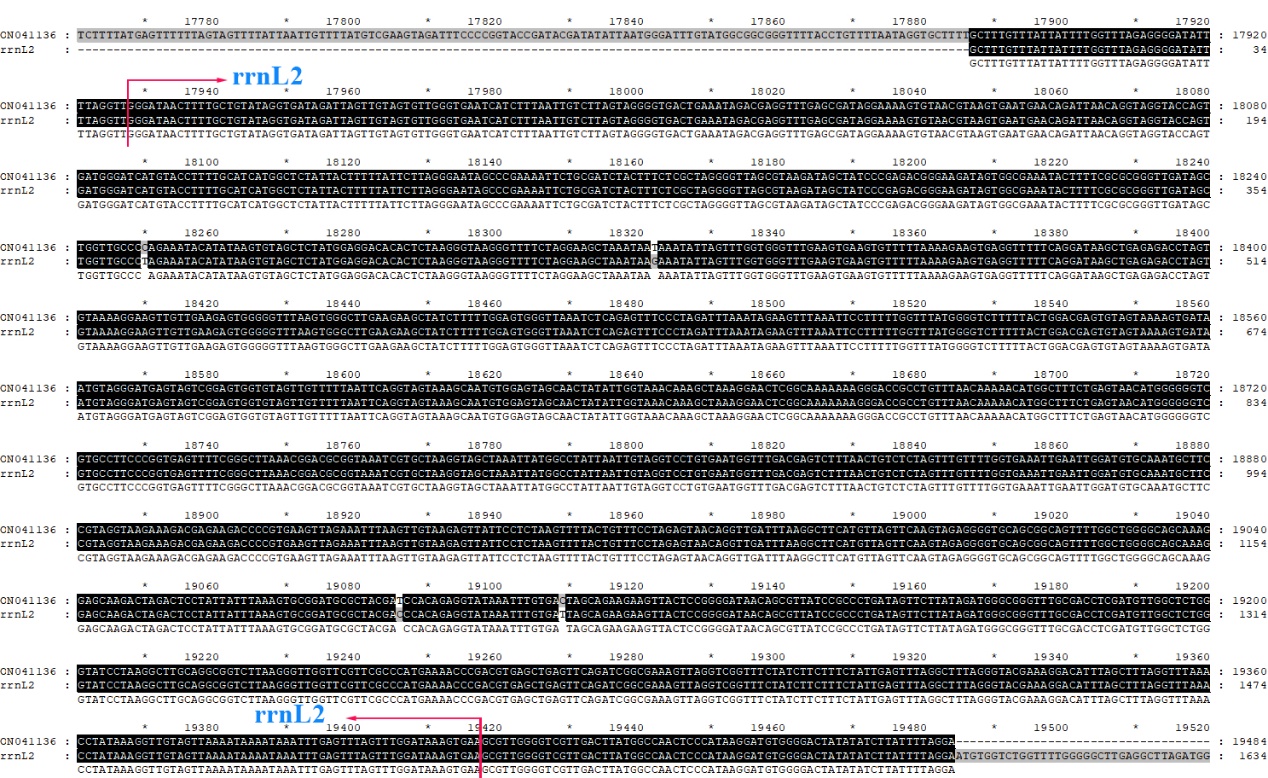


**Supplementary Fig. S3. Validation of the genomic arrangement.** The Sanger sequence from an amplicon spanning the unique junction between rrnL1 and rrnL2 aligns perfectly with the assembly, confirming their contiguous arrangement and providing definitive evidence for a genuine duplication.


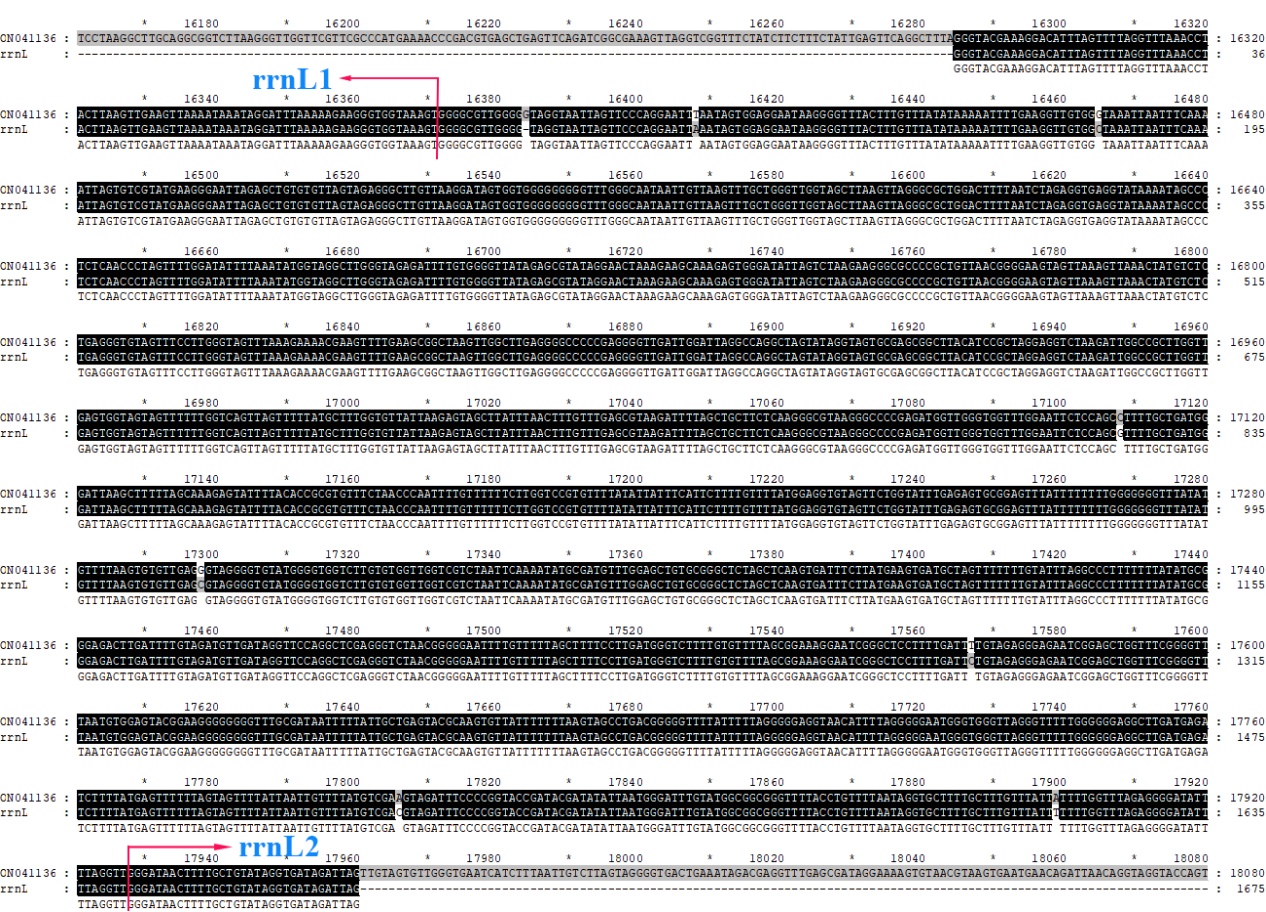


**Supplementary Fig. S4.** Coverage depth plot of the assembled mitochondrial genome of Y. balloti (GenBank accession number: ON041136). The horizontal axis represents the nucleotide position, and the vertical axis represents the read mapping depth.


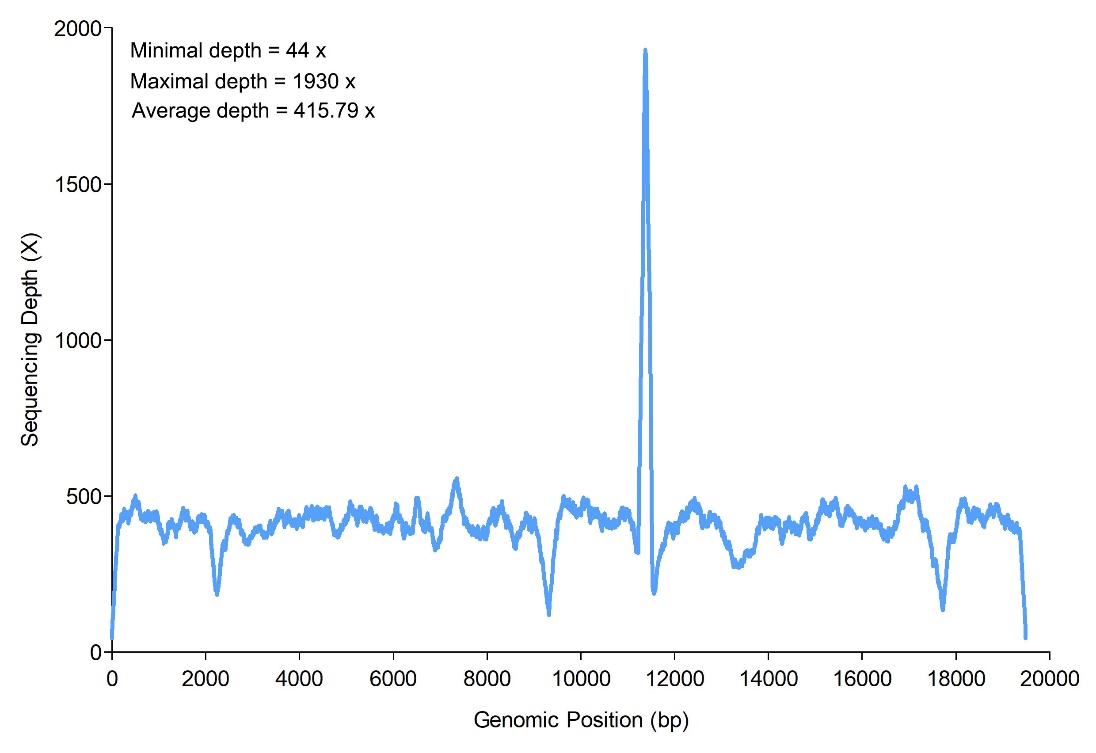


**Supplementary Table 1.** Primer sequences used for PCR validation of the duplicated 16S rRNA genes in *Y. balloti*. All primers were designed based on the assembled mitochondrial genome (ON041136).

| **Primer pair** | **Forward primer** | **Reverse primer** | **Target region** |
| --- | --- | --- | --- |
| rrnL1-1 | AAGGGAGTAGGTGTTACAAT | TACCTTAGCACGATTTACCG | Region of rrnL1 |
| rrnL1-2 | ATGAGTAGTCGGAGTGGTGT | AATTCCCTTCATACGACACT | Region of rrnL1 |
| rrnL2-1 | ATTTGTATGGCGGCGGGTTT | TTCTTACCTACGGAAGCATT | Region of rrnL2 |
| rrnL2-2 | GCGGTAAATCGTGCTAAGGT | TGGCAACCACATACCAGGAC | Region of rrnL2 |
| rrnL-1 | GGGTACGAAAGGACATTTAG | TGGGTTAGAAACACGCGGG | Intergenic region between rrnL1 and rrnL2 |
| rrnL-2 | TGGTTTGGAATTCTCCAGCC | TAATCTATCACCTATACAGC | Intergenic region between rrnL1 and rrnL2 |
